# Nutrition education intervention improves medical students' dietary habits and their competency and self-efficacy in providing nutrition care: A pre, post and follow-up quasi-experimental study

**DOI:** 10.3389/fnut.2023.1063316

**Published:** 2023-03-02

**Authors:** Bright Yammaha Amoore, Patience Kanyiri Gaa, Anthony Amalba, Victor Mogre

**Affiliations:** ^1^Department of Health Professions Education and Innovative Learning, School of Medicine, University for Development Studies, Tamale, Ghana; ^2^Department of Nutritional Sciences, School of Allied Health Sciences, University for Development Studies, Tamale, Ghana

**Keywords:** nutrition education, dietary habits, self-efficacy, competencies, medical students

## Abstract

**Objective:**

Most doctors and medical students report inadequate competencies in nutrition care. We evaluated the impact of a nutrition education intervention on medical students' lifestyle habits, dietary diversity, nutrition care knowledge, attitude toward nutrition care, and their level of self-efficacy in the provision of nutrition care.

**Methods:**

All 2nd-year medical students were enrolled into a 5 week, 24-h nutrition education intervention that involved both deductive and practical sessions. Pre-, post and 4 weeks follow-up assessments were conducted.

**Results:**

At post- and 4-weeks post-intervention the number of days participants consumed vegetables and engaged in moderate-to-vigorous physical activity significantly (*p* = 0.003 and 0.002) improved respectively from baseline. Mean nutrition care knowledge scores of participants increased by 3.27 points (95% Cl: 1.98–4.56, *p* < 0.001) from 19.49 at baseline through to 24.78 post- and 22.76 4 weeks follow-up. No significant [$X_{\left(2\right)}^{2}$ = 1.568, *p* = 0.457] change in mean attitude toward nutrition care score was recorded. Mean level of self-efficacy in the provision of nutrition care improved significantly by 1.73 (95% Cl: 1.17–2.28, *p* < 0.001) at post-intervention and 4 weeks follow-up compared to the baseline scores.

**Conclusion:**

The intervention improved the nutrition care knowledge, self-efficacy in the provision of nutrition care as well as medical students' own consumption of vegetables, dietary diversity and their engagement in moderate-to-vigorous physical activity. However, continuous implementation of nutrition education interventions is needed to sustain these outcomes and further improve the nutrition education experience of medical students.

## Introduction

Nutrition is a cost-effective approach to the prevention and management of diseases (1). Malnutrition in all its forms is the global leading cause of poor health outcomes. Nutrition-related conditions account for not less than a quarter of all primary health care visits (2, 3). The adoption of healthy dietary and lifestyle by individuals is known to reduce the occurrence of these non-communicable diseases.

Doctors are the main drivers of the health care system who doubles as the major source of referral to other relevant health care professionals (4). The general public frequently identifies doctors as one of the most trustworthy sources of nutrition information (5). They are ubiquitously reported as the most preferred source of nutrition information by clients (3). Doctors themselves recognize nutrition care to be a significant part of their practice. There is also evidence that they are potentially effective at improving the dietary and lifestyle of individuals they counsel. For example, a Behavioral Risk Factor Surveillance System study found that there is nearly 3-fold increase in patients attempts to address their nutrition disorders following a doctor's advice (6). In order to utilize these opportunities to bring about behavior changes, there is the need for doctors to have the required nutrition care competencies for their routine clinical practice (7). However, doctors frequently are unable to effectively provide nutrition care due to lack of nutrition counseling training, inadequate knowledge and low confidence and skills to give nutrition care (8, 9). Nutrition education is inefficiently incorporated into medical schools (10). Often practicing doctors, medical students, and incoming interns report being under-trained and unwilling to counsel patients with nutritional problems due to low level of medical nutrition education (11, 12). It is thus not surprising that doctors are not able to adequately diagnose and provide requisite nutrition care to clients (2, 6) leading to nutrition related diseases often going unidentified and untreated in many health care settings (13).

A number of interventions has been implemented to improve nutrition education in the medical curriculum toward promoting effective provision of nutrition care. Some of these interventions are either stand-alone courses or nutrition syllabi integrated into the medical curriculum to improve nutrition education during training. In-service training nutrition training programs have also been implemented to improve the nutrition care competencies of practicing medical doctors. The majority of these interventions are designed and implemented in developed countries (e.g., US, UK, Australia, Netherlands, etc.) that have different settings and educational environments (14–26). Given that Ghana and other African countries face similar challenges of inadequate nutrition education and poor provision of nutrition care by medical doctors (27, 28), it is necessary to design context-specific interventions to improve the provision of nutrition care by doctors. However, interventions of this nature are limited in these settings.

There is evidence to show that doctors who follow healthy dietary habits are more likely to provide nutrition care to their clients (20, 29, 30). Thus, nutrition education interventions should thus aim at not only improving medical students' nutrition care competencies but should be interested in improving their dietary and lifestyle habits.

We designed a nutrition education intervention aimed at improving the nutrition care competencies and dietary habits of Ghanaian medical students. The findings of the intervention will provide evidence on the potential benefits associated with integrating nutrition education into the medical curriculum. It will also serve as a source of reference for research in developing countries providing useful guidelines for future nutrition intervention studies in countries that have settings and educational environments similar to Ghana. We thus report here the effect of a nutrition education intervention on lifestyle habits, dietary diversity, nutrition-related competency, and self-efficacy in the provision of nutrition care among medical students.

## Methods

### Study design, setting, and participants

A pre-post and 4-weeks follow-up quasi-experimental design without control group was adopted for the study. A quasi-experimental design was used due to our inability to perform randomization due to ethical considerations, difficulty to randomly place participants into groups or locations, limited available sample size and difficulty in finding a comparable control group (31). It was also difficult randomizing the students of the selected class given that it was not possible to prevent the control group interacting with the intervention group (32). As published elsewhere (33), this study was conducted at the University for Development Studies School of Medicine in Ghana that runs a problem-based learning, community-based education and service (PBL-COBESS) curriculum for undergraduate medical education. As described elsewhere (34), the PBL/COBES curriculum is executed through system-based course modules or blocks in which teaching and learning are organized through PBL tutorials in small groups, didactic lectures, clinical skills and laboratory practical sessions during the first 3 years of preclinical training. Coordinated discipline-based clerkship rotations are adopted for the next years of clinical training. Although nutrition is usually taught in the preclinical years, opportunities for learning nutrition are very few and inadequate (28, 35). Participants were 2nd year medical students (*n* = 93) who enrolled in the 2019–2020 academic year and have no formal nutrition background. All the 93 2nd year medical students were recruited into the study in March 2021.

### Nutrition education intervention

We implemented an intervention dubbed Nutrition Life Style and Behavior Change Training for Students (NLBCTS) (33) from April through to May 2021. The NLBCTS design was informed by the recommendations reported by Lindsley et al. (36) in which they identified nutrition care competencies that medical students should develop. It covered the following thematic areas: nutrition throughout the life cycle, nutrition and health, food nutrients, nutrition assessment, malnutrition in children, nutrition and non-communicable diseases, patient counseling and motivational interviewing, nutrition in health promotion and disease prevention and referral to registered dieticians. It was informed by the findings of a previous review that identified what, how and why nutrition education interventions work to improve nutrition care competencies and provision of nutrition care (37). The intervention was incorporated into an ongoing course module in which free periods were utilized. Each of the sessions lasted for 2 h. The NLBCTS was conducted over a 5-week period, totaling 24 contact hours. The intervention was designed to supplement the increasing demand of nutrition education by medical students in an attempt to promote safe and effective nutrition health care practices by physicians (38). Students were trained using multiple teaching and learning activities including interactive lecture presentations using PowerPoint, demonstrations, problem-based learning tutorials, tasting sessions, nutrition games, and role plays. The multiple learning activities aimed to meet the demand of students with varying learning approaches to promote easy assimilation of the intervention in a way that will increase the likelihood of students to apply the acquired skills in their future practice (39). The innovative approach was markedly focused on the skills and attitude development of students to cultivate competence that promote self-efficacy and nutrition counseling behavior (37). The study also tapped from the social cognitive learning theory which propounded that, behaviors are learned by observation and outlined important learning processes that are necessary for maintaining goal-oriented behaviors.

### Recruitment and data collection procedures

Information was sent to students through their WhatsApp and other social media platforms to recruit them into the intervention. Using paper-based questionnaires, data was collected at three time points i.e., at baseline, post-intervention and 4 weeks follow-up assessment (conducted at the end of June 2021). Students were provided with information about the intervention and informed consent was signed before participation. We encouraged voluntary participation, assured and ensured confidentiality of their responses.

### Data collection methods

Lifestyle and dietary habits of the students were also measured. These were related to consumption of breakfast, fried foods, fruits and vegetables, snacking in between meals, alcohol intake and participation in moderate- to vigorous-intensity physical activity for at least 30 min. For each of these, students were asked to indicate the number of days they ate those foods or engaged in those lifestyle behaviors in the last 7 days.

The dietary diversity of the students was also assessed using the FAO guidelines for measuring household and individual dietary diversity (40). Dietary diversity scores reflects nutrient adequacy of the diet (40). Students were asked to describe the foods (meals and snacks) that they ate or drank in the past 24 h (day and night) whether at their residence or elsewhere. The food items recalled by the students were then classified into a list of 12 food groups depending on the information provided. The food groups were cereals, white tubers and roots, vegetables, fruits, meat, fish and other seafood, eggs, legumes, nuts and seeds, milk and milk products, oils and fats, sweets, and spices, condiments, and beverages. Responses of the students to each of the food groups were used to generate dietary diversity scores (41). The dietary diversity score was obtained by summing the number of unique food groups consumed by each student.

We also assessed students' nutrition care competencies (including knowledge and attitudes) and their level of self-efficacy in the provision of nutrition care. Nutrition care knowledge was measured using twenty-eight (28) questions. Out of this number, eleven (11) were multiple-choice questions and four (4) where questions of the true or false format. The remaining questions had either one or more correct answers. These questions assessed students' knowledge of basic concepts in nutrition and their application to nutrition care. They were derived from the intervention topics and previously validated studies (22, 38). A wrong answer attracted zero (0) score and a correct answer was scored one (1). The total expected score was 52. Attitude toward nutrition care was assessed using a 9-item questionnaire in which responses were made on a 5-point Likert scale ranging from 1-strongly disagree to 5-strongly agree. Total scores were generated and weighted to range between 1 and 5. The items were derived from previously validated questionnaires (38, 42). A 9-item scale was used to evaluate students' level of self-efficacy in the provision of nutrition care in which responses ranged from 1- very uncomfortable to 10-very comfortable. The items were derived from previously published studies that found to be valid and reliable (22) and surveys (43–45). Total self-efficacy scores were computed for each student and weighted to range between 1 and 10.

### Data analysis

Data was entered into and analyzed using SPSS software version 21. Descriptive statistics of mean, standard deviation and frequencies were used to describe the data. Normality test was conducted using Shapiro Wilk test to determine appropriate test techniques for data analysis. Repeated measures and Friedman tests were applied to analyze parametric and non-parametric data respectively to evaluate the difference in test score for the pre, post and 4-weeks follow up assessment. The significance level was < 0.05 at 95% confidence interval. With an effect size of 0.4 for nutrition care knowledge and at a sample size of 93, the study had a power of 0.96.

### Ethical consideration

Ethical clearance for the research was sought from the Committee on Human Research, Publication and Ethics of the Kwame Nkrumah University of Science and Technology. This was proceeded with a permission request from the Dean of the School of Medicine to conduct the study among students of the school. Students who agreed to participate were taken through the consent procedures and were assured that participation was voluntary.

## Results

### General and socio-demographic characteristics of the participants

Ninety-three students were recruited into the study in which 64% (*n* = 59) were male students. At 4-weeks follow-up, one student differed the programme and was lost to follow (age = 18 years, male) and was subsequently excluded from the analysis. Also, 1–6 participants skipped all the items used to assess the outcomes either at baseline, post-intervention or 4-weeks follow-up (refer to their respective tables for details) and were excluded from the analysis for their respective outcomes. Less than half of the study participants (44.6%, *n* = 41) reported to have a choice of medical specialty, from which 30.4% (*n* = 14) preferred cardiothoracic surgery, 17.4% (*n* = 8) neurology and 15.2 (*n* = 7) general surgery. Participation rates in all activities of the intervention ranged from 80 to 100%.

### Lifestyle and dietary habits

Table 1 shows the results of Friedman test on the baseline, post-intervention and 4 weeks' post-intervention measurements for dietary and lifestyle habits of the study participants.

**Table 1:** Baseline, post- and 4-weeks follow-up mean number of days participants participated in lifestyle habits.

**Dietary and lifestyle habit**	**Baseline**	**Post**	**4 weeks follow-up**	***p*** **-value**	**η^2^**
Number of days of taking breakfast (*n* = 91)	3.51 (2.24)	3.96 (2.07)	4.23 (5.74)	0.128	0.02
Skipping of breakfast (*n* = 89)	3.43 (2.18)	2.83 (2.06)	3.11 (2.06)	0.142	0.14
Number of days snacks are consumed in between meals (*n* = 90)	2.59 (2.34)	2.59 (1.99)	2.76 (2.02)	0.381	0.01
Vegetable consumption (*n* = 89)	2.36 (2.13)	3.16 (2.05)	2.69 (2.13)	0.003	0.07
Fruit consumed (*n* = 86)	1.51 (1.82)	1.65 (1.50)	1.81 (1.79)	0.309	0.01
Fried foods consumed (*n* = 87)	3.11 (2.15)	3.37 (1.90)	3.29 (1.84)	0.299	0.01
Alcohol intake (*n* = 88)	0.16 (0.79)	0.28 (0.93)	0.50 (1.26)	0.032	0.03
Engagement in moderate- to-vigorous physical activity for at least 30 min (*n* = 88)	1.77 (2.14)	2.40 (2.02)	2.14 (2.01)	0.002	0.07

Varying number of participants for each of the variables is due to participants skipping those items either at baseline, post-intervention and 4 weeks follow-up. These were excluded from the analysis for the respective variables.

The mean number of days participants consumed vegetables per week increased significantly to 3.16 (95% Cl, 2.72–3.59, *p* = 0.001) days from 2.36 (95% Cl, 1.92–2.80) days at baseline. This decreased to 2.69 (95% Cl, 2.23–3.14) 4-weeks post-intervention (*Z* = −2.344, *p* = 0.019) but still higher than the baseline scores. As shown in Table 1 the mean number of days participants engaged in moderate-to-vigorous physical activity for at least 30 min per week increased significantly from 1.77 (95% Cl: 1.32–2.23) to 2.40 (95% Cl: 1.97–2.83, *p* = 0.002) days post-intervention and decreased slightly to 2.14 (95% Cl: 1.17–2.56, *p* = 0.248) days 4-weeks post-intervention. No significant (*Z* = −1.792, *p* = 0.073) change in the mean number of days was observed between the 4 weeks post-intervention score and the baseline.

### Dietary diversity

Table 2 presents the results of the Friedman test on the pre-intervention, post-intervention and retention measurements of change in dietary diversity scores of study participants. The proportion of students who ate food from the individual foods groups at baseline, post-intervention and 4 weeks follow-up (retention) did not differ significantly. However, the mean (SD) total dietary diversity scores of the participants increased from 5.00 (1.35) at baseline to 5.28 (1.29) post intervention (*Z* = −1.439, *p* = 0.150) and to 4.87 (1.37) at 4 weeks post intervention (*Z* = −2.516, *p* = 0.012). But the decline was not statistically significant compared to the baseline (*Z* = −0.359, *p* = 0.720).

**Table 2:** Baseline, post-intervention and 4 weeks follow-up mean dietary diversity and frequency of consumption of food groups among the participants compared using Friedman test.

**Food item**	**Baseline**	**Post**	**4 weeks follow-up**	***p*** **-value**
Cereals (*n* = 84)	83 (98.8)	83 (98.8)	82 (97.6)	0.779
White tubers and roots (*n* = 84)	21 (25.0)	27 (32.1)	24 (28.6)	0.509
Vegetables (*n* = 84)	75 (89.3)	65 (77.4)	67 (79.8)	0.085
Fruit (*n* = 83)	8 (9.6)	11 (13.3)	5 (6.0)	0.293
Meat (*n* = 84)	19 (22.6)	29 (34.5)	19 (22.6)	0.072
Egg (*n* = 84)	21 (25.0)	28 (33.3)	18 (21.4)	0.139
Fish (*n* = 84)	10 (11.9)	13 (15.5)	7 (8.3)	0.325
legume nuts and seeds (*n* = 84)	33 (39.3)	36 (42.9)	38 (45.2)	0.703
Milk and milk products (*n* = 84)	8 (9.5)	4 (4.8)	9 (10.7)	0.291
Oils and fats (*n* = 84)	73 (86.9)	68 (81.0)	72 (85.7)	0.485
Sweets (*n* = 84)	0 (0.0)	3 (3.6)	3 (3.6)	0.449
Spices, beverages (*n* = 84)	66 (78.6)	70 (83.3)	64 (76.2)	0.469
**Total dietary diversity (*****n*** **=** **83)**	**5.00 (1.35)**	**5.28 (1.29)**	**4.87 (1.37)**	**0.038**

For the total dietary diversity, 3 participants skipped all the items at baseline and six skipped at 4-weeks follow-up and were excluded from the analysis.

### Nutrition care competency and self-efficacy to provide nutrition care

Table 3 shows the mean nutrition care knowledge, attitude toward nutrition care and level of self-efficacy in the provision of nutrition care of the participants.

**Table 3:** Baseline, post- and 4-weeks follow-up mean nutrition knowledge, attitude and self-efficacy scores of participants compared using repeated measurement test.

**Variable**	**Baseline**	**Post**	**4-weeks follow-up**	***p*** **-value**	**η^2^**
Nutrition-related knowledge (*n* = 92)	19.49 (3.77)	24.78 (4.93)	22.76 (4.89)	<0.001	0.40
Attitude toward the provision of nutrition care (*n* = 90)	4.21 (0.48)	4.09 (0.74)	4.03 (0.69)	0.457	0.01
Level of self-efficacy in the provision of nutrition care (*n* = 89)	5.30 (1.97)	7.02 (1.35)	6.44 (1.44)	<0.001	0.30

Two students skipped all the items that assessed attitude toward nutrition care and were excluded from the analysis for attitude. Three participants skipped all the items for the self-efficacy variable either at baseline, post-intervention or 4-weeks follow-up and were excluded from the analysis for self-efficacy.

The mean nutrition care knowledge scores of the students increased significantly from 19.49 (95% Cl: 18.71–20.27) at pre-intervention to 24.78 (95% Cl: 23.76–25.80) immediately after the intervention (*Z* = 5.293, 95% Cl: 4.115–6.472, *p* < 0.001) and decreased to 22.76 (95% Cl: 23.77–21.75) 4-weeks follow-up (*Z* = 2.02, 95% Cl: 0.86–3.19, *p* < 0.001). Despite the decline, the 4 weeks post-intervention score was significantly higher than the baseline score (*Z* = 3.272, 95% Cl: 1.98–4.56, *p* < 0.001). Mean attitude toward nutrition care score at baseline was 4.21 (95% Cl: 4.11–4.31), 4.09 (95% Cl: 3.93–4.24) immediately after the intervention and 4.03 (95% Cl: 3.88–4.17) 4-weeks follow-up. The differences were not statistically significant (*p* = 0.457).

Also, the study participants experienced a significant improvement in mean (SD) levels of self-efficacy in the provision of nutrition care from 5.30 (1.97) at baseline to 7.02 (1.33) post-intervention (*Z* = 1.725, 95% Cl: 1.17–2.28, *p* < 0.001), and to 6.44 (1.44) at 4 weeks post intervention (*Z* = 0.59, 95% Cl: 0.33–0.84, *p* < 0.001). But the mean level of self-efficacy in the provision of nutrition care at 4-weeks follow-up remained significantly higher than the mean score at baseline (*Z* = 1.14, 95% Cl: 0.56–1.72, *p* < 0.001).

## Discussion

The study aimed to investigate the effect of a nutrition education intervention on the lifestyle habits and dietary diversity of medical students as well as their nutrition care competencies, and self-efficacy in the provision of nutrition care. The 24 contact hour, 5 week NLBCTS intervention resulted in a modest improvement in students' own lifestyle and dietary habits in terms of vegetable consumption, participation in moderate-to-vigorous physical activity, and dietary diversity. It also improved medical students' nutrition care knowledge and their self-efficacy in the provision of nutrition but maintained the already positive attitude students had toward the provision of nutrition care. However, 4-weeks post-intervention assessment showed a slight decline in the improvement of the outcomes but not lower than the baseline values.

Our current study found a significant improvement in vegetable consumption among medical students after the intervention. The intervention also improved the dietary diversity scores of the medical students demonstrating that the students ate more diversified diets after the intervention. This is similar to the findings of previous studies that reported significant improvement in dietary habits such as fruit intake, increased consumption of homemade food among restaurant and pre-prepared meals, avoidance of fatty foods, increased frequency of wholegrain food intake and decreased consumption of processed meat, after following a nutrition education intervention (14, 19–21, 24, 25, 46, 47).

Physical activity is one of the key fundamentals to good health outcomes (48). Previous studies (29, 30) found that doctor and medical students' engagement in physical activity is associated with regional increase in physical activity among a population of US adults which exemplifies favorable attitude toward preventive counseling by medical students. The significant improvement in moderate-to-rigorous physical activity recorded by this study is consistent with the findings of Tavolacci et al. (30) where medical students' engagement in physical activity increased due to frequent practice of sports. The improvement in the personal lifestyle and dietary habits of the students demonstrates their likelihood of providing nutrition care as previous studies (20, 29, 30) have reported that, doctors or future medical doctors who follow healthy dietary habits are often more likely to provide similar care to their clients. Thus, the ability of the intervention to improve some of the lifestyle and dietary habits of students will not only impact their personal wellbeing but may also be passed onto their future clients (18, 19).

In line with previous studies the NLBCTS significantly improved medical students' knowledge to enable them provide nutrition care (18, 19, 22). Although, there was a decline in the nutrition-related knowledge scores of the students' 4 weeks post intervention, the scores remained higher than the baseline scores. This finding is consistent with the findings of Berz et al. (15) who reported a slight decline in the 4-week follow-up scores but higher than the knowledge scores at baseline among a sample of medical students that participated in interactive nutrition education sessions during an ambulatory medicine rotation. It was also found that the significantly higher mean post-intervention score of 24.78 (95% Cl: 23.76 to 25.80) and the 4 weeks post-intervention score of 22.76 (95% Cl: 21.75 to 23.77) were below 50% denoting a below average score of the total nutritional knowledge score of 52. The low or suboptimal improvement in nutrition-related knowledge score found by the study is similar to what was reported by Coppoolse et al. (22) among a sample of medical students in the Netherlands in which the nutrition education intervention improved nutrition-related knowledge but the improved scores were below average.

Evidently, medical students generally perceive nutrition care as one of the fundamental roles of medical doctors (49). The current study buttresses this fact given that the already existing positive attitude and motivation to give nutrition care shown by the medical students at baseline was sustained throughout the lifespan of the intervention as confirmed by the 4-weeks follow-up assessment. This is similar to those of previous studies (19, 50).

The study recorded a significant change in medical students' level of self-efficacy in the provision of nutrition care. Much of this improvement was recorded immediately after the intervention and retained at 4-weeks follow-up. This finding is similar to the findings of Coppoolse et al. (22). The increase in self-efficacy is a demonstration of students' increased ability, confidence and awareness to provide nutrition care and support to individuals to adopt healthy dietary and lifestyle habits as reported by Crowley et al. (21). Additionally, the improved self-efficacy score demonstrates the ability of the intervention to position participants to give nutrition care in their future medical practice if the current level of self-efficacy is sustained throughout their medical training. This can only be possible if more opportunities are created in the curriculum to continue to provide nutrition education to the students. There is evidence that student's familiarity with evidence-based nutrition interventions and understanding of the role of interprofessional engagement further enhance their efficacy to address lifestyle-related illness (25).

The study has a number of strengths worth noting. The response rate of 100% to the study was very high compared to those of previous nutrition education interventions (14, 25). Also, the study used a standardized questionnaire for assessing the knowledge, attitude, self-efficacy and preparedness of participants. The three time-point assessment (baseline, post-intervention and 4-weeks follow-up) conducted by the study allows for assessing longitudinal impact of the intervention on participants (14). Additionally, the post-intervention evaluation conducted by the study to gather students' perspectives or feedback concerning the effectiveness of the study reported elsewhere afforded the opportunity for strengthening future interventions. By way of implications, it is evident that a 5-week nutrition education intervention is capable of improving the nutrition related-knowledge, attitude, self-efficacy, lifestyle and dietary habits, and preparedness of medical students to provide nutrition care in their future medical practice. The findings suggest that the sustained level of improved self-efficacy of students throughout the study, as well as the improved nutrition-related knowledge and already existing positive attitude toward nutrition care among the participants, highlight the intervention's ability to increase students' ability, confidence, awareness, and preparedness to provide nutrition care and also support individuals to adopt healthy dietary and lifestyle habits. The improved lifestyle and dietary habits of participants coupled with their preparedness to provide nutrition care as reported elsewhere (33), also illustrate that nutrition education interventions if well-designed could improve the likelihood of students to provide nutrition care in their future practice. The 4-week follow-up assessment that showed a slight decline in a number of the primary outcomes of the intervention demonstrate the need for providing opportunities for students to consolidate and reinforce the competencies that have been realized till they graduate. Previous studies have had similar suggestions and recommendations regarding the integration of nutrition education throughout the curriculum (22, 51). There is evidence that the integration of nutrition education throughout the curriculum is effective at improving effective provision of nutrition care by doctors (37).

This study is not without limitations. Firstly, the survey items were self-reported making the findings liable to recall and social-desirability biases. Moreover, the study focused on pre-clinical year and did not cover clinical year students which is a crucial phase of their professional development noted to be characterized with unhealthy lifestyles due to heavy workload and emotional stress (52), which can negatively impact on the attitude and overall preparedness of participants to give nutrition care in their future practice (18, 19, 29, 30). There is thus the need for future research to be scaled-up to students in the clinical year. Research in this direction will further afford the opportunity to compare the outcome of nutrition education in these groups. Finally, because the study is from one medical school in Ghana it will be difficult to generalize the findings to the entire country. The findings however may inform the design of similar studies in other parts of the country and beyond.

## Conclusion

The intervention demonstrates an effective strategy for improving medical students' lifestyle and dietary habits. The NLCBCTS can also help medical students improve their nutrition care competencies. Opportunities for reinforcement to consolidate gains made should be provided, as the level of improvement in most of the outcomes of the intervention decreased slightly at the 4-week follow-up assessment, despite remaining higher than baseline scores.

## Data availability statement

The original contributions presented in the study are included in the article/supplementary material, further inquiries can be directed to the corresponding author.

## Ethics statement

The studies involving human participants were reviewed and approved by Committee on Human Research, Publications and Ethics of the Kwame Nkrumah University of Science and Technology. The patients/participants provided their written informed consent to participate in this study.

## Author contributions

BA and VM jointly conceived and designed the study together. BA collected and performed data analysis and interpretation and drafting of manuscript. VM, PG, and AA jointly undertook critical revision of the manuscript. VM gave the final approval of the version to be published. All authors agree to be accountable for all aspects of the work.
